# Deciphering the microbiota compositions of complex biofilms from hospital and domestic settings across Estonia, Germany, and the United Kingdom

**DOI:** 10.1093/femsle/fnaf118

**Published:** 2025-10-23

**Authors:** Christina Killian, Guerrino Macori, Isabella Centeleghe, Peeter Laas, Rebecca Lewis, Nicole van Leuven, Katie Wall, Razan Abbara, Ralf Lucassen, Marja Hagström, Dirk Bockmuehl, Mark Fielder, Noora Perkola, Veljo Kisand, Jean-Yves Malliard, Séamus Fanning

**Affiliations:** UCD-Centre for Food Safety, University College Dublin, Dublin D05 N2E5, Ireland; UCD School of Biology and Environmental Science, University College Dublin, Dublin D05 N2E5, Ireland; UCD-Centre for Food Safety, University College Dublin, Dublin D05 N2E5, Ireland; UCD School of Biology and Environmental Science, University College Dublin, Dublin D05 N2E5, Ireland; School of Pharmacy and Pharmaceutical Sciences, Cardiff University, Cardiff CF10 3NB, United Kingdom; Institute of Technology, Tartu University, Nooruse 1, Tartu 50411, Estonia; School of Life Sciences, Pharmacy and Chemistry, SEC Faculty, Kingston University London, Kingston Upon Thames, KT1 2EE, United Kingdom; Rhine-Waal University of Applied Sciences, Faculty of Life Sciences, Kleve, D-47533, Germany; UCD-Centre for Food Safety, University College Dublin, Dublin D05 N2E5, Ireland; UCD School of Public Health, Physiotherapy and Sports Science, University College Dublin, Dublin D05 N2E5, Ireland; School of Life Sciences, Pharmacy and Chemistry, SEC Faculty, Kingston University London, Kingston Upon Thames, KT1 2EE, United Kingdom; Rhine-Waal University of Applied Sciences, Faculty of Life Sciences, Kleve, D-47533, Germany; Finnish Environment Institute, Latokartanonkaari 11, 00790 Helsinki, Finland; Rhine-Waal University of Applied Sciences, Faculty of Life Sciences, Kleve, D-47533, Germany; School of Life Sciences, Pharmacy and Chemistry, SEC Faculty, Kingston University London, Kingston Upon Thames, KT1 2EE, United Kingdom; Finnish Environment Institute, Latokartanonkaari 11, 00790 Helsinki, Finland; Institute of Technology, Tartu University, Nooruse 1, Tartu 50411, Estonia; School of Pharmacy and Pharmaceutical Sciences, Cardiff University, Cardiff CF10 3NB, United Kingdom; UCD-Centre for Food Safety, University College Dublin, Dublin D05 N2E5, Ireland; UCD School of Public Health, Physiotherapy and Sports Science, University College Dublin, Dublin D05 N2E5, Ireland

**Keywords:** biofilm, next-generation sequencing, *One Health*

## Abstract

Currently, there is a limited understanding of the microbiota composition of complex biofilms, in particular describing the abundance of bacterial genera, fungi, and yeasts with reference to the *One Health* axes. As a starting point, describing the microbiota found in these settings would begin to describe the nature of any biological hazards present and facilitate development of strategies to limit transmission and the spread of infection. Furthermore, using this approach, suitable interventional measures could then be tested in the laboratory-scale model for their efficacy and then applied *in situ*. COMplex Biofilms and AMR Transmission (COMBAT) is a consortium of research teams that studied the application of next-generation sequencing (NGS) strategies to identify bacterial, fungal, and yeast species present in selected biofilms recovered from drain settings found in domestic and hospital settings in four geographical regions. Findings from this study extended our understanding of the bacterial, fungal, and yeast abundances in these sample biofilms and how they may change following enrichment. A VSEARCH-based high-resolution clustering approach was implemented to full-length 16S rRNA (FL-16S) sequencing reads to generate near-ASV operational units, enabling detailed characterization of the microbial communities within complex biofilms. This analytical framework provides the basis for testing interventional measures at the laboratory scale that could be implemented to reduce risk to the *One Health* axes.

## Teaser

In this study, a novel *in vitro* biofilm model, mimicking a drain trap, and next-generation sequencing, were employed to characterize the bacterial, fungal, and yeast populations within drain biofilms.

## Introduction

Bacteria exist in an individual, planktonic state, where they can undergo chemotaxis to locate and use nutrients essential for survival (Adler 1966). However, once a suitable environment is found, these genera of bacteria may come together, with other microbes, to form a biofilm.

Effective biocides are important for the management of biofilms in some economically relevant ecological niches, such as across the food chain and in health care settings (Cámara et al. 2022). The European Union *One Health* 2023 Zoonoses report highlighted some 229 food-borne outbreaks of campylobacteriosis with 148 181 confirmed human cases, 1115 food-borne outbreaks of salmonellosis with 77 486 confirmed human cases, 66 food-borne outbreaks of Shiga toxin-producing *Escherichia coli* infections with 10 217 confirmed human cases and 19 food-borne outbreaks of listeriosis with 2952 confirmed human cases ((EFSA) and (ECDC) 2024). In addition, contaminated drinking water supplies can lead to legionnaires’ disease, caused by *Legionella* species (Brouwer et al. 2024). In hospital settings, contact surfaces located in moist environments with available nutrients, may present an opportunity for colonization of diverse genera of bacteria that can come together to form biofilms. The first clinical biofilm-associated infections were found to be device-related infections. In 1982 Marrie et al. (1982), using scanning electron microscopy, found the inner surface and internal wires of a pacemaker lead were coated with heavy biofilms of *Staphylococcus aureus*. Outbreaks linked to hospital drainage systems continue to be reported and are causing significant challenges—in particular hospital-acquired infections (Inkster 2024). It is becoming more apparent that biofilm drain samples from residential, as well as hospital settings, harbour multidrug-resistant organisms (Hayward et al. 2025), and these are challenging to eradicate using common disinfectants (Hennebique et al. 2025). Accurate identification and subsequent surveillance of resistant bacterial pathogens in these settings would facilitate the directed administration of appropriate therapy, as well as limiting the spread of infection and aiding control measures in hospital settings (Ajulo and Awosile 2024, Sauerborn et al. 2024).

Next-generation sequencing (NGS) technologies have revolutionized the exploration of microbial diversity in complex environments such as biofilms (Nafea et al. 2023). In particular, amplicon-based approaches targeting conserved regions like the 16S ribosomal RNA (rRNA) gene are widely used to profile bacterial communities. Short-read sequencing of hypervariable regions (e.g. V3–V4) provides taxonomic data at genus level, while long-read technologies enable full-length 16S (FL-16S) rRNA gene analysis, supporting more accurate species-level classification. These methods also extend to the detection of fungal and yeast communities through sequencing of the internal transcribed spacer (ITS) region, albeit with caution due to intragenomic variability (Bradshaw et al. 2023).

Despite these advances, the microbial composition of biofilms, particularly in environments that intersect human, animal, and environmental health, remains poorly characterized. Given the protective matrix and the mixed-species nature of biofilms, standard cultivation methods often fail to capture their full diversity. In contrast, NGS-based approaches offer a powerful solution to address this gap by enabling culture-independent profiling of microbial communities. This study aimed to characterize and compare the taxonomic composition of complex biofilms derived from domestic and hospital drains across multiple European regions.

**Figure 1.**
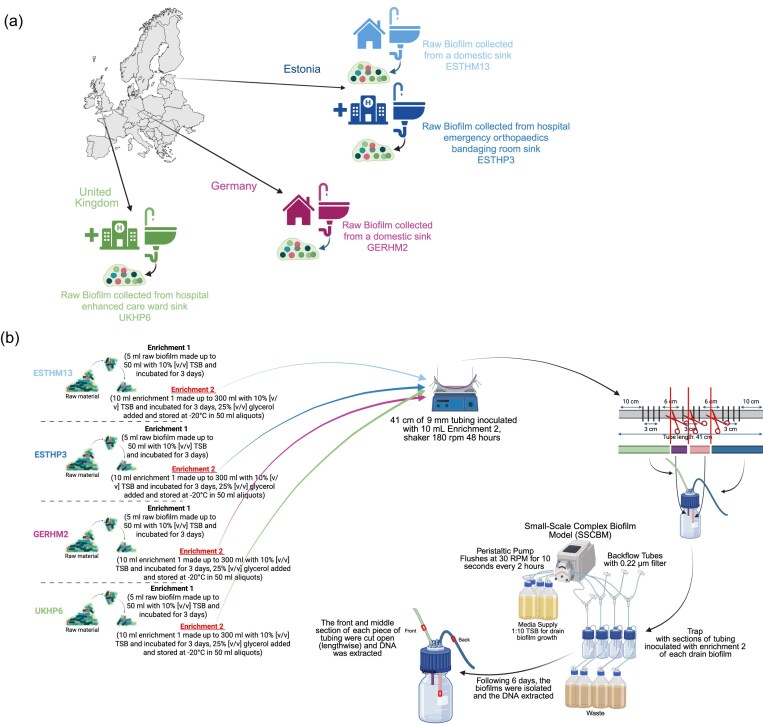
(a) Schematic representation of the raw biofilms and where they originated. One biofilm from a hospital drain was collect from the UK (colour coded green), another biofilm from a home bathroom drain was collected from Germany (colour coded pink), and two biofilms were collected from Estonia—one from a hospital drain (colour coded dark blue) and another from a home bathroom drain (colour coded light blue). (b) Illustration of the SSCBM. An *in vitro* biofilm model for testing complex drain biofilms and the efficacy of disinfection, developed by Ledwoch et al. (2020). Figures were created with BioRender.

A novel *in vitro* biofilm model, mimicking a drain trap, small-scale complex biofilm model (SSCBM), developed previously by Ledwoch et al. (2020), was employed to characterize the bacterial, fungal, and yeast populations within drain biofilms (Fig. 1). NGS strategies were used to explore the microbiota of each distinct biofilm community. The findings provide a comparative description of diversity and potential health relevance in these niches across different geographical and environmental contexts. The described methods include a novel VSEARCH-based amplicon sequence variants (ASV) approach to FL-16S rRNA gene sequencing reads. This approach could in turn, be used to assess interventional measures and applied to mitigate the formation of biofilms.

## Material and methods

### Biofilm sample collection

In total, 86 distinct *in situ* biofilms were collected from COMBAT partners spanning the three *One Health* areas: 28 hospitals, 22 home samples, and 36 farm samples. For this study, four ‘core’ raw biofilms were chosen to be taken forward and applied to the SSCBM. These were taken from geographically and environmentally distinct locations across Europe, including domestic (bathroom drain) and hospital (ward drain) environments as part of the COMBAT study (Table 1). Samples originated from one domestic and one hospital setting in Estonia (the latter from an emergency orthopaedics bandaging room drain); one domestic drain in Germany; and one hospital ward drain (enhanced care unit) in the UK (Fig. 1a). The U-bend trap of each sink drain was unscrewed and a bucket was placed underneath to avoid water dripping. A sterile spatula was used to remove slurry of drain to ~10 ml of 50 ml falcon tube—this equates to ~4.5 g, which was required for freeze cultures. Once the falcon tube was sealed properly, the U-bend trap of each sink drain was reassembled and checked to ensure there were no leaks. The time of collection and sample code was recorded on each falcon tube containing sample. Upon receipt in the laboratory at UCD, all falcon tube samples were stored at –20°C prior to further processing.

**Table 1.**
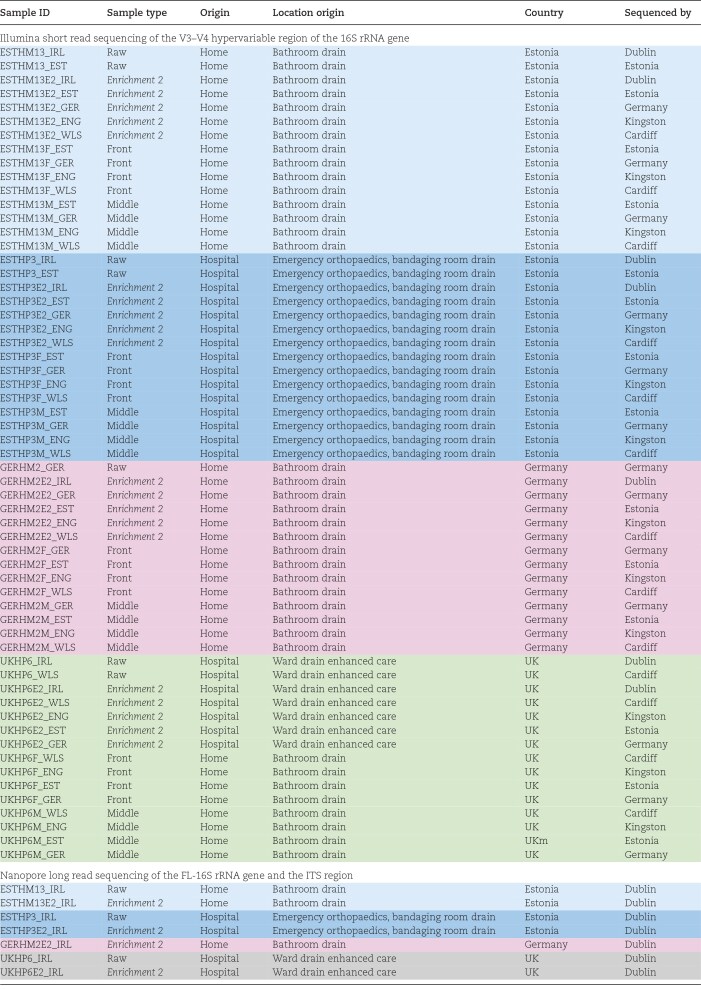
A table providing an overview of raw and enriched biofilm samples collected from domestic (bathroom drain) and hospital (ward drain) environments across four European countries (Estonia, Germany, the UK, and Ireland) as part of the COMBAT study. Samples were either analysed in their raw state, after Enrichment 2 (denoted as E2) or after passing through the SSCBM—E2 isolated from the front (F) or middle (M) section of the SSCBM—and were processed by different partner laboratories. The sample identifier (ID) follows the SSCBM structure, where prefixes denote origin, setting, and biofilm ID number (e.g. *ESTHM13* = Estonia Home biofilm ID 13 raw), and suffixes indicate the sequencing site or institution (e.g. *_IRL* = sequenced in Dublin, *_ENG* = sequenced in Kingston). This standardized coding supports clear cross-institutional tracking of sample processing and sequencing. 59 samples were subjected to short read 16S rRNA amplicon sequencing. Seven samples that were only sequenced in Dublin were subjected long-read 16S rRNA amplicon sequencing and ITS analysis to explore fungal diversity (see also Fig. S1).

### Microbiota enrichment

To enhance the detection of culturable microbiota within biofilm matrices, each sample was subjected to two enrichment steps prior to long-term storage. Enrichment 1 was generated by diluting 5 ml of raw biofilm in 10% (v/v) tryptic soy broth (TSB) to a final volume of 50 ml, followed by static incubation at room temperature for 72 h. Subsequently, 10 ml of Enrichment 1 was transferred into 300 ml of fresh 10% (v/v) TSB (or 10 parts Phosphate Buffered Saline (PBS)) to create Enrichment 2, which was also incubated under the same conditions. After incubation, 25% (v/v) glycerol was added to the Enrichment 2 culture for cryopreservation. Aliquots (50 ml) were stored at –20°C for subsequent analyses.

### Genomic DNA purification from biofilm samples and sequencing

Total genomic DNA (gDNA) was extracted directly from the raw biofilm samples using standard protocols optimized for environmental samples. Microbial community profiling was conducted using NGS targeting: (i) the V3–V4 hypervariable region of the 16S rRNA gene; (ii) the FL-16S rRNA gene; and (iii) the ITS region for fungal and yeast identification (Fig. S1). These sequencing approaches enabled taxonomic resolution from genus to species level across bacterial and fungal domains.

In brief, raw biofilm samples and their corresponding Enrichment 2 counterparts were thawed on ice, and a volume of 1 ml of each sample centrifuged at 18 000 × *g* for 5 min to pellet biomass. gDNA was extracted using the Invitrogen™ PureLink™ Genomic DNA Mini Kit following the manufacturer’s instructions with minor modifications to enhance yield from environmental matrices. Pelleted material was resuspended in 180 µl Genomic Digestion Buffer and treated with 20 µl proteinase K (20 mg/ml), followed by incubation at 55°C for 4 h with intermittent vortexing every 15–30 min until complete lysis. RNase A (20 µl) was then added and incubated at room temperature (21°C) for 2 min. After vortexing with 200 µl genomic lysis/binding buffer and 200 µl of ethanol (96%–100% v/v), samples were transferred to spin columns and centrifuged to bind DNA. Wash steps included sequential application of 500 µl of wash buffer 1 and wash buffer 2, followed by elution with 100 µl of genomic elution buffer.

DNA concentration was measured using a Qubit® 2.0 Fluorometer, and quality was assessed using a Nanodrop^®^ ND-1000 Spectrophotometer. DNA yields ranged from 1.99 to 207.00 ng/µl. A minimum concentration of 10 ng/µl was required for Illumina short-read sequencing, and ~0.67 ng/µl was required for FL-16S sequencing with the ONT platform.

### Amplicon-based 16S rRNA gene sequencing

To investigate the bacterial communities, both short-read and long-read 16S rRNA gene sequencing approaches were applied. For short-read sequencing, two raw biofilm samples (from Estonia and UK) and all four Enrichment 2 samples (from Estonia, UK, and Germany) were sent to Novogene (or eurofins in Konstanz, Germany—depending on country of sequencing) for commercial sequencing targeting the V3–V4 region of the 16S rRNA gene. Due to different sequencing providers and locations—we do expect a degree of variation across biofilm samples. For long-read sequencing, the same samples were processed using the ONT MinION Mk1B platform. Amplification of the FL-16S rRNA gene was performed using the ONT 16S Barcoding Kit 24 V14 (SQK-16S114.24), starting from 10 ng gDNA. Barcoded libraries were pooled and loaded onto FLO-FLG114 Flongle Flow Cells and sequenced for 24 h.

### ITS amplicon sequencing for fungal and yeast communities

To characterize fungal and yeast components of the same biofilms, ITS sequencing was performed using the primer pair ITS1-F_KYO2 (5′-TAG AGG AAS TAA AAG TCG TAA-3′) and LR5 (5′-TCC TGA GGG AAA CTT CG-3′). Each 12.5 µl Polymerase Chain Reaction (PCR) reaction consisted of 6.25 µl LongAmp™ Hot Start Taq 2X Master Mix (New England Biolabs), 0.5 µl of each primer, 10 ng template gDNA, and nuclease-free water. Reactions were assembled in low-binding 96-well plates, mixed gently, centrifuged at 1000 × *g* for 1 min, and amplified using the following thermal cycling conditions: 95°C for 3 min; 25 cycles of 95°C for 15 s, 55°C for 15 s, and 72°C for 30 s.

Amplicons were subsequently verified using the Agilent 2200 TapeStation with D5000 ScreenTape reagents to confirm the expected ∼1500 bp product size. After validation, amplicons were barcoded and prepared using the ONT Native Barcoding Kit 96 V14 (SQK-NBD114.96), pooled at a final concentration of 10–20 fmol, and loaded onto FLO-FLG114 Flongle Flow Cells for 24-h sequencing runs.

### Bioinformatic analysis

#### Illumina short-read analysis

Quality control of Illumina paired-end reads (V3–V4 region of the 16S rRNA gene) was performed using FastQC v0.12.1, followed by filtering, trimming, error correction, dereplication, and ASV inference using the DADA2 pipeline (Callahan et al. 2016) in *RStudio* v2024.09.0 + 375. Chimeric sequences were removed during the DADA2 workflow. The resulting ASV table was taxonomically annotated using the SILVA v138 reference database. Downstream analyses, including alpha- and beta-diversity calculations, were performed using the phyloseq and vegan packages in *RStudio*. Ordinations (PCoA) were computed based on Bray–Curtis distances to explore differences in microbial community structure.

#### Nanopore long-read analysis

For FL-16S and ITS Nanopore reads, raw FAST5 files were basecalled and demultiplexed using Dorado, Oxford Nanopore Technologies basecalling toolkit. Reads were further filtered for quality using NanoFilt (De Coster et al. 2018) to remove low-quality and short reads. Taxonomic classification was performed using Kraken2 with: (i) SILVA v138 database for 16S rRNA gene sequences; and (ii) UNITE v9.0 database for fungal ITS reads. Classification outputs were imported into *RStudio* for aggregation, abundance filtering, and visualization using phyloseq. Diversity metrics (e.g. Shannon and Simpson), taxonomic bar plots, and ordination analyses were produced to compare microbial profiles across different sample types, origins, and enrichment stages. Wilcoxon, Kruskal–Wallis, and Bonferroni *post hoc* statistical tests were also applied.

#### Comparative ASV high-resolution clustering from long reads using VSEARCH

In parallel, a comparative pipeline was applied to a subset of the nanopore FL-16S reads using VSEARCH v2.15.2, with the aim of performing high-resolution clustering independently of Kraken2-based classification to improve taxonomic resolution.

This pipeline involved: (i) concatenation of filtered full-length reads from selected samples into a single FASTA file; (ii) dereplication of reads and removal of singletons; (iii) clustering sequences at a stringent identity threshold of 99.5% (–id 0.995) using –usearch_global; and (iv) mapping of reads to the clustered ASVs to generate an Operational Taxonomic Unit(s) (OTU)-style abundance table (–otutabout); comparison with Kraken2 results to assess consistency of taxa detected and estimate differences in diversity and relative abundance between classification methods. Jobs were executed on a high-performance computing system using SLURM-managed parallel jobs.

## Results

### Sample biofilm selection and processing

Biofilm samples were collected from hospital and domestic drains across Estonia, Germany, and the UK. Each raw biofilm and its corresponding Enrichment 2 (denoted as E2), as well as front (F) or middle (M), was subjected to template gDNA extraction. Front (F) or middle (M) samples are E2 cultures, which have been passed through the SSCBM and isolated from the front (F) or middle (M) section of the SSCBM, (see Fig. 1b). Concentrations of purified gDNA ranged from 1.99- to 207-ng/µl. gDNA quality and yield were suitable for sequencing, with ≥10 ng/µl required for short-read 16S amplicon sequencing (Illumina, V3–V4) and ~0.67 ng/µl for long-read FL-16S sequencing (Oxford Nanopore, FL-16S). This enabled comparison of microbial diversity and composition across raw and enriched biofilms (Table 1).

Short-read 16S amplicon sequencing yielded a total of 1 338 635 high-quality reads, ranging from 1329 to 103 616 reads per sample. One sample (GERHM2_GER) yielded only five reads and was excluded from downstream analysis (denoted blank in Fig. 3c). FL-16S sequencing yielded 2 254 717 reads, ranging from 62 465 to 567 238 reads. Both sequencing strategies highlighted the taxonomic diversity across sample types and regions. Rarefaction analyses (Fig. S2) confirmed the fact that sufficient sequencing depth for robust alpha diversity comparisons has achieved.

### Revealing the taxonomic complexity of biofilm samples

**Figure 2.**
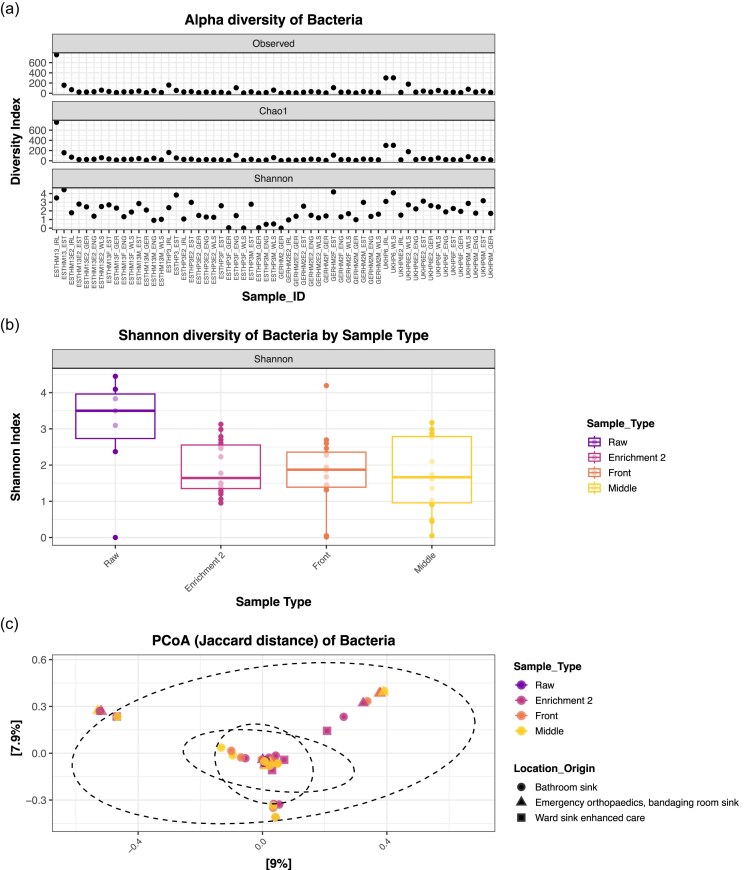
Diversity as a comparison of the raw and enriched biofilms. (a) Alpha diversity illustrated by Observed, Chao1, and Shannon indices for raw, enriched front, and middle biofilms from 16S rRNA amplicon-mediated sequencing of the V3–V4 polymorphic region. (b) Shannon diversity of raw, enriched front, and middle biofilms from 16S rRNA amplicon-mediated sequencing of the V3–V4 polymorphic region. Statistical significance was calculated using Kruskal–Wallis rank sum test. (c) Principal coordinate analysis using the Jaccard similarity test examining the similarity between the microbiota compositions of the different biofilms. Data clustered for 16S rRNA amplicon-mediated sequencing of the V3–V4 polymorphic region. The ellipse indicates shared microbial taxa between samples.

Alpha diversity was assessed using Observed species, Chao1 richness, and Shannon diversity indices (Fig. 2). For short-read 16S data (Fig. 2a), most samples had consistent richness across indices. However, samples such as UKHP6_WLS, ESTHM13_EST, and ESTHM13_IRL displayed elevated richness and evenness. Boxplot analysis of Shannon diversity revealed that raw biofilms were more diverse than Enrichment 2 (E2), Front (F), and Middle (M) samples, although not significant (*P*-value = .086) (Fig. 2b). This suggests that enrichment may reduce community complexity, possibly due to selective growth conditions favouring specific taxa. PCoA based on Jaccard distances (Fig. 2c) demonstrated clustering of raw samples across regions, while E2, F, and M biofilms appeared more variable in community composition. Jaccard distance was applied as it was the most robust approach particularly when relative abundances may be biased by sequencing conditions.

### Taxonomic profiles and regional trends obtained using short-read sequencing

**Figure 3.**
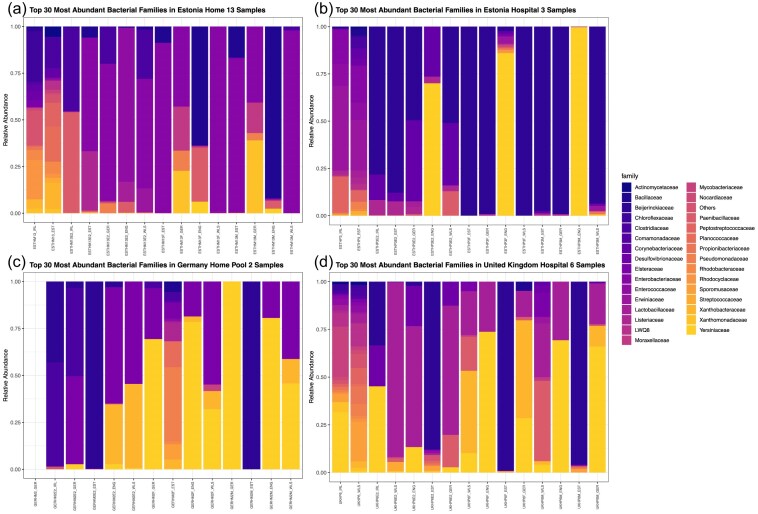
A stacked bar chart plot showing the top 30 abundance families for (a) raw, E2, M, and F Estonia Home 13 complex biofilm samples, (b) raw, E2, M, and F Estonia Hospital 3 complex biofilm samples, (c) raw, E2, M, and F Germany Home pool 2 complex biofilm samples, and (d) raw, E2, M, and F United Kingdom Hospital 6 complex biofilm samples.

Taxonomic classification of microbial communities was visualized through stacked bar plots at the family level (Fig. 3), revealing distinct patterns associated with location, biofilm source (hospital versus home), and treatment condition (raw versus enrichment versus front versus middle).

### Domestic drain biofilms from Estonia

Raw biofilms obtained from household bathroom drains (ESTHM13_IRL, ESTHM13_EST) consistently featured *Clostridiaceae*, reinforcing their role as core members of the environmental microbiome in these domestic settings (Fig. 3a). *Enterococcaceae* was a prevalent family in many of the enriched biofilms and those samples post-SSCBM from the front and middle sections, derived from these raw biofilms (e.g. ESTHM13E2_EST, ESTHM13E2_GER, ESTHM13E2_ENG, ESTHM13E2_WLS, ESTHM13F_EST, ESTHM13F_GER, ESTHM13F_WLS, ESTHM13M_EST, ESTHM13M_GER, and ESTHM13M_WLS) though its absence in others (e.g. ESTHM13E2_IRL, ESTHM13F_ENG, and ESTHM13M_ENG) highlights the variability introduced during enrichment and post-SSCBM. This suggests certain families may be more susceptible to enrichment dynamics or influenced by subtle interlaboratory procedural differences (Fig. 3a).

### Hospitals drain biofilms from Estonia

In the raw biofilm samples collected from hospital drains in Estonia (ESTHP3_IRL, ESTHP3_EST), the dominant families identified by sequencing were *Lactobacillaceae* and *Enterococcaceae*, both indicative of a complex, native microbiota (Fig. 3b). However, following the samples sequenced in Kingston (ENG), the taxonomic profile shifted markedly, with *Yersiniaceae* becoming dominant (Fig. 3b). The shift in profile suggests that the enrichment process selected for fast-growing facultative anaerobic taxa and potentially displaced more sensitive or slow-growing community members. Differences between enriched samples processed in different locations (e.g. Estonia versus Dublin or Cardiff) further suggest that storage or transport conditions may have contributed to community restructuring.

### Domestic drain biofilms from Germany

Biofilm samples from German home environments, such as GERHM2E2_GER, community structure changed from enriched to post-SSCBM. Before being applied to the SSCBM, GERHM2E2_GER, consisted mainly of *Clostridiaceae* and *Enterobacteriaceae*, however, post-SSCBM the persistence of *Xanthomonadaceae* and *Yersiniaceae* across these samples (Fig. 3c). As sample GERHM2_GER yielded only five reads, this was illustrated by a white box and was excluded from downstream analysis (Fig. 3c).

### Hospital drain biofilms from the UK

Raw biofilms collected from UK hospital drains (UKHP6_WLS, UKHP6_IRL) were dominated by *Listeriaceae, Pseudomonadaceae*, and *Yersiniaceae* (Fig. 3d). These families include many opportunistic pathogens and environmental generalists, aligning with the hospital setting’s potential for harbouring stress-tolerant species. Following enrichment, *Bacillaceae, Enterobacteriaceae, Lactobacillaceae*, and *Yersiniaceae* remained prominent across nearly all replicates, suggesting resilience or adaptability to *in vitro* growth conditions (Fig. 3d). The maintenance of these taxa may reflect their ecological versatility or potential to form biofilm substructures resistant to change during enrichment.

### Revealing bacterial family complexity of biofilm samples using long-read sequencing

**Figure 4.**
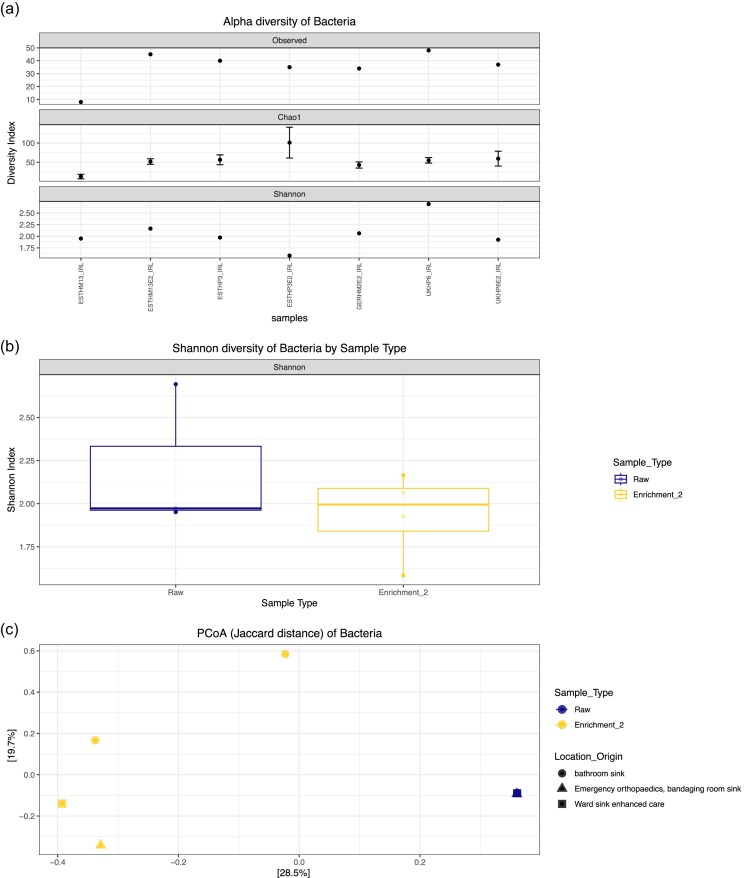
Diversity as a comparison of the raw and enriched biofilms (a) Alpha diversity illustrated by Observed, Chao1, and Shannon indices for raw and enriched biofilms from FL-16S rRNA gene sequencing. (b) Shannon diversity of raw and enriched biofilms from FL-16S rRNA gene sequencing. Statistical significance was calculated using Wilcoxon rank-sum exact test. (c) Principal coordinate analysis using the Jaccard similarity test examining the similarity between the microbiota compositions of the different biofilms. Data clustered for FL-16S rRNA gene sequencing.

Alpha diversity was assessed using Observed species, Chao1 richness, and Shannon diversity indices (Fig. 4). For long-read FL-16S data (Fig. 4a), most samples had consistent richness across indices. However, UKHP6E2_IRL showed the highest species richness and diversity, while ESTHP3E2_IRL showed the lowest. Boxplot analysis of Shannon diversity revealed that raw biofilms were more diverse than E2 samples, although not significantly (*P*-value = .63) (Fig. 4b). As stated previously, this suggests that enrichment may reduce community complexity, possibly due to selective growth conditions favouring specific taxa.

PCoA based on Jaccard distances (Fig. 4c) demonstrated clustering of raw samples across regions, while enriched biofilms appeared more variable in community composition. There was a clear distinction between raw and enriched biofilms, with raw samples clustering tightly and enriched samples appearing dispersed, indicating enrichment substantially alters community structure (Fig. 4c).

### Taxonomic profiles and geographic regional trends observed using long-read sequencing

**Figure 5.**
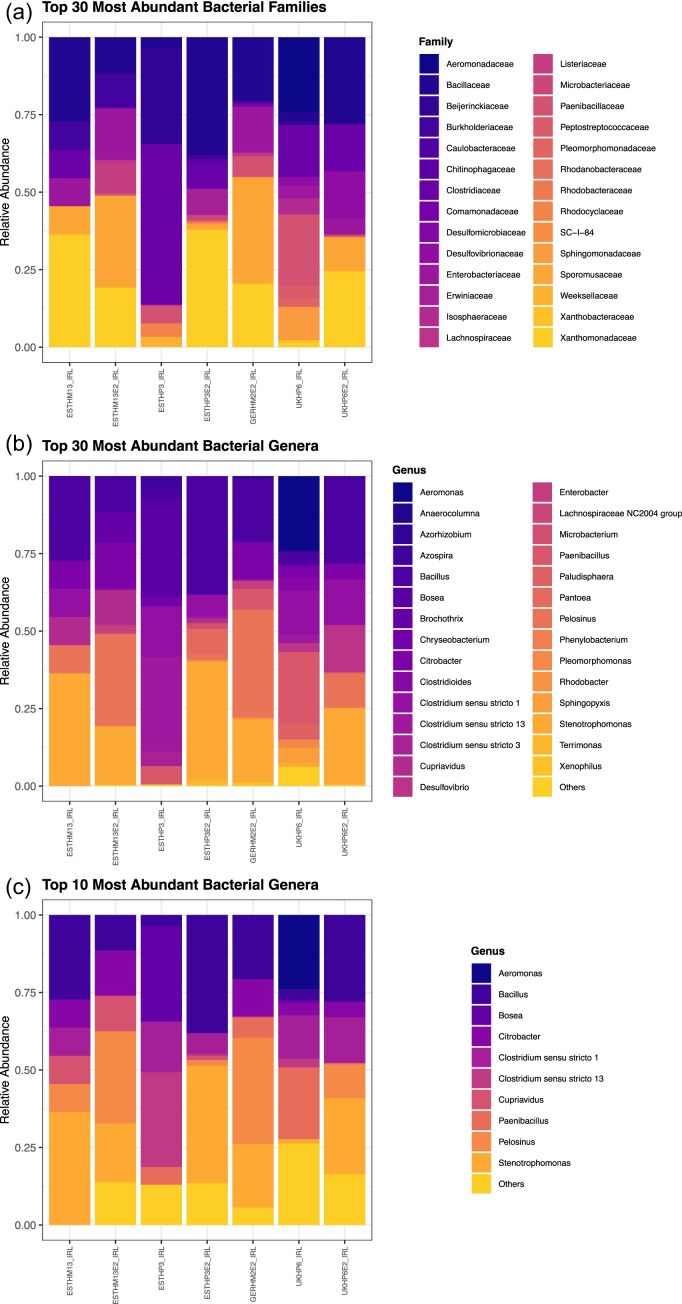
A stacked bar chart plot showing the (a) 30 most abundant families, (b) 30 most abundant genera, and (c) top 10 abundant genera from FL-16S rRNA gene sequencing of complex biofilms.

Taxonomic classification of microbial communities was visualized through stacked bar plots at the family and genus levels (Fig. 5), revealing distinct patterns associated with geography, biofilm source (hospital versus home), and treatment condition (raw versus enrichment). All biofilms exhibited similar relative abundance profiles with *Aeromonas* present only in UKHP6_IRL and *Clostridium* mainly present in GERHM2E2_IRL (Fig. 5c).

### Insights from FL-16S rRNA gene sequencing

Analysis of FL-16S rRNA amplicons using the same sample set as above, confirmed *Clostridiaceae* as a core taxon across all samples, underscoring its widespread prevalence in drain biofilms irrespective of geography, enrichment, or sequencing strategy. *Stenotrophomonas* was detected in all samples except GERHM2E2_IRL, highlighting their overall persistence but also suggesting occasional loss or suppression under certain enrichment or processing conditions. Of particular note, *Citrobacter* was consistently identified by sequencing in both raw and enriched samples from UK hospital drains, further validating this family’s ecological stability across sequencing approaches. Among all samples, UKHP6E2_IRL was notable as the most diverse by Shannon index (Fig. 4a), suggesting a uniquely rich and evenly distributed community, possibly due to its location in a high-contact (frequent touch contact surface(s) by staff/patients), high-nutrient (contains biofilm, moisture, and organic residues) hospital environment—resulting in a high-risk hotspot where microbes survive well and are easily transferred due to frequent human interaction.

### Long-read ASV clustering using VSEARCH reveals greater resolution in biofilm community structure

ASVs were inferred from FL-16S rRNA reads to allow comparisons between short- and long-read sequencing. ASVs are typically used for short-read sequencing and detect sequence variations at a single-nucleotide level. OTUs are generally used for long-read sequencing and are clustered based on a similarity threshold—typically 97% identity. VSEARCH clustering provided enhanced taxonomic resolution compared to both short-read ASV (Illumina V3–V4) and OTU-based long-read approaches. Across the samples analysed, the long-read ASVs captured a broader range of unique taxa, particularly within complex and heterogeneous communities such as the UK hospital biofilms and Estonian household drains. In several cases, clusters previously grouped under the same OTU were resolved into distinct ASVs, uncovering microheterogeneity that was not detected using full-length OTUs alone. Comparative analysis showed that while both raw and enriched samples maintained major taxa such as *Clostridium sensu stricto, Bacillus*, and *Stenotrophomonas*, the ASV-based profiles displayed finer distinctions between sample origins, especially in identifying less abundant or niche genera (Fig. 5c). Notably, ASVs derived from long-read data exhibited greater evenness and richness in raw biofilms than in enriched counterparts, reinforcing earlier diversity trends seen in short-read results, but with added precision. These findings validate the utility of long-read ASV clustering for environmental biofilm studies, supporting its potential for improved pathogen surveillance and *One Health* applications.

### ITS amplicon-mediated gene sequencing

As a proof of principle that other taxonomic components of biofilms beyond bacteria can be detected in a biofilm, the presence of fungi and yeast were investigated through targeted sequencing of the nuclear ribosomal ITS region. Nanopore long read sequencing of the same sample sets as described above, produced in total 1 439 698 high quality reads ranging from 12 186 (UKHP6_IRL) to 1 292 530 (ESTHM13E2_IRL) (Fig. S2).

**Figure 6.**
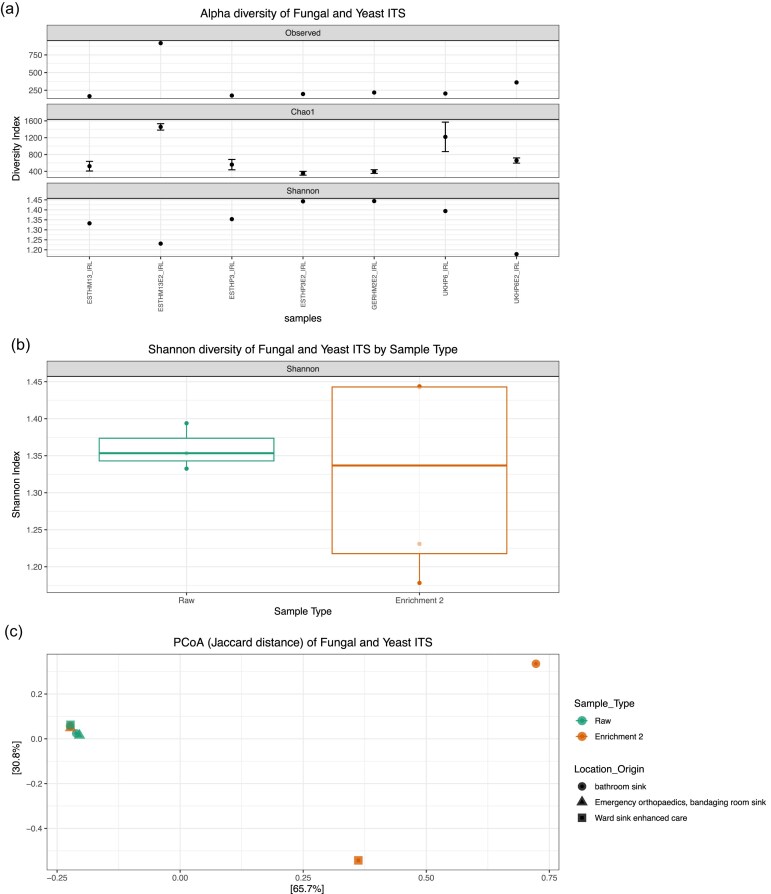
Diversity as a comparison of the raw and enriched biofilms. (a) Alpha diversity illustrated by Observed, Chao1, and Shannon indices for raw and enriched biofilms from ITS amplicon-mediated gene sequencing. (b) Shannon diversity of raw and enriched from ITS amplicon-mediated gene sequencing. Statistical significance was calculated using Wilcoxon rank-sum exact test. (c) Principal coordinate analysis using the Jaccard similarity test examining the similarity between the microbiota compositions of the different biofilms. Data clustered for ITS amplicon-mediated gene sequencing.

The alpha diversity was examined to determine the spread of abundances and the level of diversity across each biofilm. Observed and Chao1 indices showed that all biofilms had similar numbers of observed species, except for samples UKHP6_IRL and ESTHM13E2_IRL, which had higher numbers of species (Fig. 6a). UKHP6_IRL had a higher number of species in the Chao1 index compared to the Observed index (Fig. 6a). The Shannon index indicated that the raw biofilms had a more even spread of abundances with ESTHP3E2_IRL and GERHM2E2_IRL being most diverse and ESTHM13E2_IRL and UKHP6E2_IRL being least diverse (Fig. 6a). Boxplot analysis of Shannon diversity revealed that raw biofilms were less diverse than E2 samples, although not significantly (*P*-value = .86) (Fig. 6b).

Principal coordinate analysis (PCoA) plots were generated showing the Jaccard test similarity between the microbiota compositions of the different biofilms (Fig. 6c). Targeted sequencing of the nuclear ribosomal ITS region showed one clear cluster, except for two outliers—both enriched biofilm samples from drain origin (Fig. 6c).

**Figure 7.**
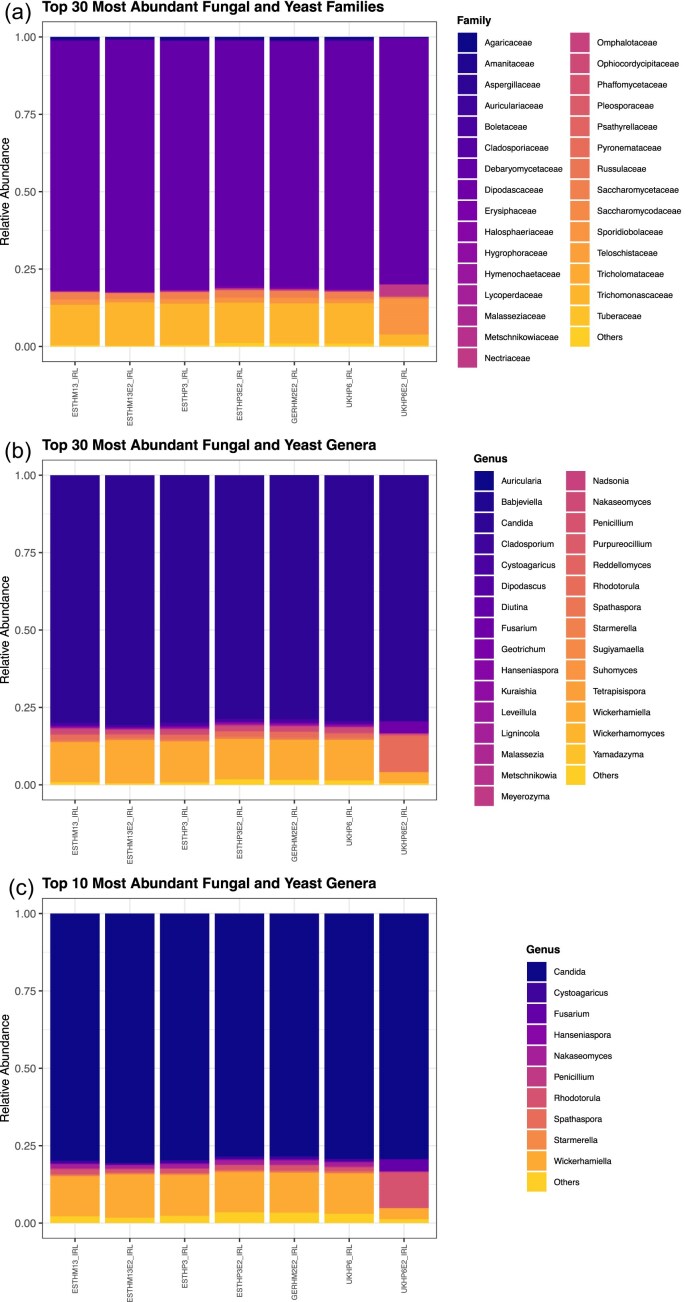
A stacked bar chart plot showing the (a) top 30 most abundance families, (b) top 30 most abundant genera, and (c) top 10 most abundant genera from targeted sequencing of the nuclear ribosomal ITS region of the complex biofilms.

Investigation of the top 30 fungal and yeast family abundances for two of the four raw complex biofilms (Estonia and UK) and all four enrichment 2 (Estonia, UK, and Germany), showed that *Debaryomycetaceae* was most abundant across all samples. UKHP6_IRL, ESTHP3_IRL, ESTHP3E2_IRL, ESTHM13E2_IRL, and GERHM2E2_IRL also showed *Trichomonascaceae* as their second most abundant family followed by *Sporidiobolaceae* and *Saccharomycetaceae*. However, UKHP6E2_IRL had *Sporidiobolaceae* as its second most abundant followed by *Nectriaceae* (Fig. 7a).

Considering abundance of the top 30 and 10 genera, *Candida* was the most abundant across all biofilms. UKHP6_IRL, ESTHP3_IRL, ESTHP3E2_IRL, ESTHM13E2_IRL, and GERHM2E2_IRL had *Wickerhamiella* as their second most abundant followed by *Rhodotorula* and *Nakaseomyces*. However, UKHP6E2_IRL had *Rhodotorula* as its second most abundant followed by *Wickerhamiella* and *Fusarium* (Fig. 7b and c).

## Discussion

COMplex Biofilms and AMR Transmission (COMBAT) is a consortium that came together to investigate the microbial communities present in biofilms from hospital and domestic drains across Europe, with sampling sites in Estonia, Germany, and the UK (Fig. 1). A novel *in vitro* biofilm model, mimicking a drain trap, called a SSCBM, developed by Ledwoch et al. (2020), was employed in this study. As each biofilm sample was sourced from a drain, and drains are a known source of microbial pathogens in healthcare settings, each biofilm sample was applied to the SSCBM to characterize the bacterial, fungal, and yeast populations within these biofilms.

This study aimed to characterize and compare the taxonomic composition of complex biofilms derived from domestic and hospital drains across multiple European regions. By integrating both short-read (V3–V4 region) and FL-16S rRNA gene sequencing strategies, we obtained a high-resolution view of microbial community structure. Our results revealed notable differences in microbial composition between raw and enriched biofilms and across geographical locations, with implications for biofilm resilience and disinfection strategies under the *One Health* framework.

ASVs are typically used for short-read sequencing and detect sequence variations at a single-nucleotide level. OTUs are generally used for long-read sequencing and are clustered based on a similarity threshold—typically 97% identity. However, in order to allow comparisons between short- and long-read sequencing, we implemented a VSEARCH-based high-resolution clustering to the FL-16S reads. This method enables discrimination of closely related taxa at single-nucleotide resolution, providing a more accurate and granular view of microbial diversity. We anticipate that ASV clustering will reveal greater microheterogeneity and core community signatures, particularly in low-abundance taxa that may be missed by OTU binning. Moreover, it will allow us to revisit subtle differences between raw and enriched biofilms with a higher taxonomic fidelity, enhancing our understanding of microbial adaptation, persistence, and potential resistance profiles in complex surface biofilms.

Diversity measures confirmed that raw biofilms harboured a greater microbial richness and evenness compared to their corresponding enrichment cultures, particularly for FL-16S sequencing. This observation suggests that cultivation or enrichment steps, while useful for isolating fast-growing or functionally relevant species, may unintentionally select against more slow-growing taxa, thereby reshaping the microbial community structure. For instance, in the Estonian hospital drain biofilm, *Bacillaceae* dominated only after enrichment, whereas these were not prominent in the corresponding raw sample. This profile shift highlights the potential for certain bacterial families to opportunistically expand when microbial competition or inhibition is reduced during enrichment. Similarly, *Yersiniaceae* was found in higher abundance in several enriched samples (e.g. ESTHP3E2_ENG), despite being less dominant in the corresponding raw biofilm and enriched biofilms, showing effects due to different sequencing providers and locations. Given recent reports linking *Bacillus* species to hospital-acquired infections (Ozkocaman et al. 2006, Mbhele et al. 2021), these findings emphasize the clinical relevance of monitoring such genera in healthcare environments (EClinicalMedicine 2021). This also highlights that biofilm manipulation, for example enrichment in this case, allows certain bacteria with potential harmful effects to thrive, such as *Bacillus*. Conversely, some raw samples (e.g. ESTHP3_EST) exhibited higher taxonomic diversity, potentially due to reduced transit times and immediate processing at the site of origin. Differences in storage, handling and sequencing site (e.g. Estonia versus Ireland) may have introduced variability in recovered taxa, underscoring the importance of consistent protocols for interlab microbiome comparisons.

The geographic regional trends noted were further supported by the family and genus-level profiles. *Lactobacillaceae, Clostridiaceae, Pseudomonadaceae*, and *Xanthomonadaceae* were associated with UK hospital biofilms, while Estonian and German home drain biofilms frequently featured *Enterococcaceae, Bacillaceae*, and *Streptococcaceae*. In several cases, enriched samples retained some key taxa from the original biofilm (e.g. *Bacillus* and *Yersiniaceae*), but the overall diversity was reduced, reflecting selective growth advantages under enrichment conditions.

In addition to bacterial profiling, targeted ITS sequencing of fungi and yeasts revealed a high abundance of *Candida*, particularly *C. auris*, a species of growing concern due to its intrinsic antifungal resistance (Kean and Ramage 2019).

This study highlights the diversity of drain biofilms across different geographical locations. The SSCBM also highlights the importance of biofilm eradiation using detergents and disinfectants, as each drain biofilm thrived within the model. A biofilm can become resistant to biocides through various means such as functional efflux pumps, changes in outer membrane structure, genetic adaptation, enzyme-mediated resistance, limited diffusion, and communication of biocides through the matrix and the level of biofilm metabolic activity (Singh et al. 2017). Accurate identification and subsequent reduction of bacterial pathogens would prevent the spread of infection and aid control measures in hospital settings (Ajulo and Awosile 2024, Sauerborn et al. 2024). The difference in diversity observed in this study could be driven not only by *One Health* but also by the selective pressure exerted by various chemicals within these biofilm communities.

## Conclusions

The COMBAT study demonstrates the utility of NGS to characterize biofilm microbiota from diverse environments within a *One Health* context. By comparing raw and enriched biofilms from hospital and domestic drains across Estonia, Germany, and the UK, we highlight both conserved and region-specific microbial profiles. Notably, the recurrent presence of clinically significant families such as *Bacillaceae* and *Pseudomonadaceae*, and the identification of *Candida auris*, underscore the potential health risks posed by persistent surface biofilms.

Our findings emphasize that biofilms are not only reservoirs of microbial diversity but also dynamic ecosystems shaped by their origin, handling, and selective pressures during enrichment. The ongoing high-resolution ASV analysis of long-read data is expected to further refine these insights, providing robust tools for surveillance and control of biofilm-associated pathogens.

## Supplementary Material

fnaf118_Supplemental_File
