# Supplementary material for: Deciphering the microbiota compositions of complex biofilms from hospital and domestic settings across Estonia, Germany, and the United Kingdom
Source: FEMS Microbiol Lett. 2025 Oct 23;372:fnaf118. doi: 10.1093/femsle/fnaf118 (PMC12604003; doi:10.1093/femsle/fnaf118)
Supplement: fnaf118_Supplemental_File [file fnaf118_supplemental_file.pdf]

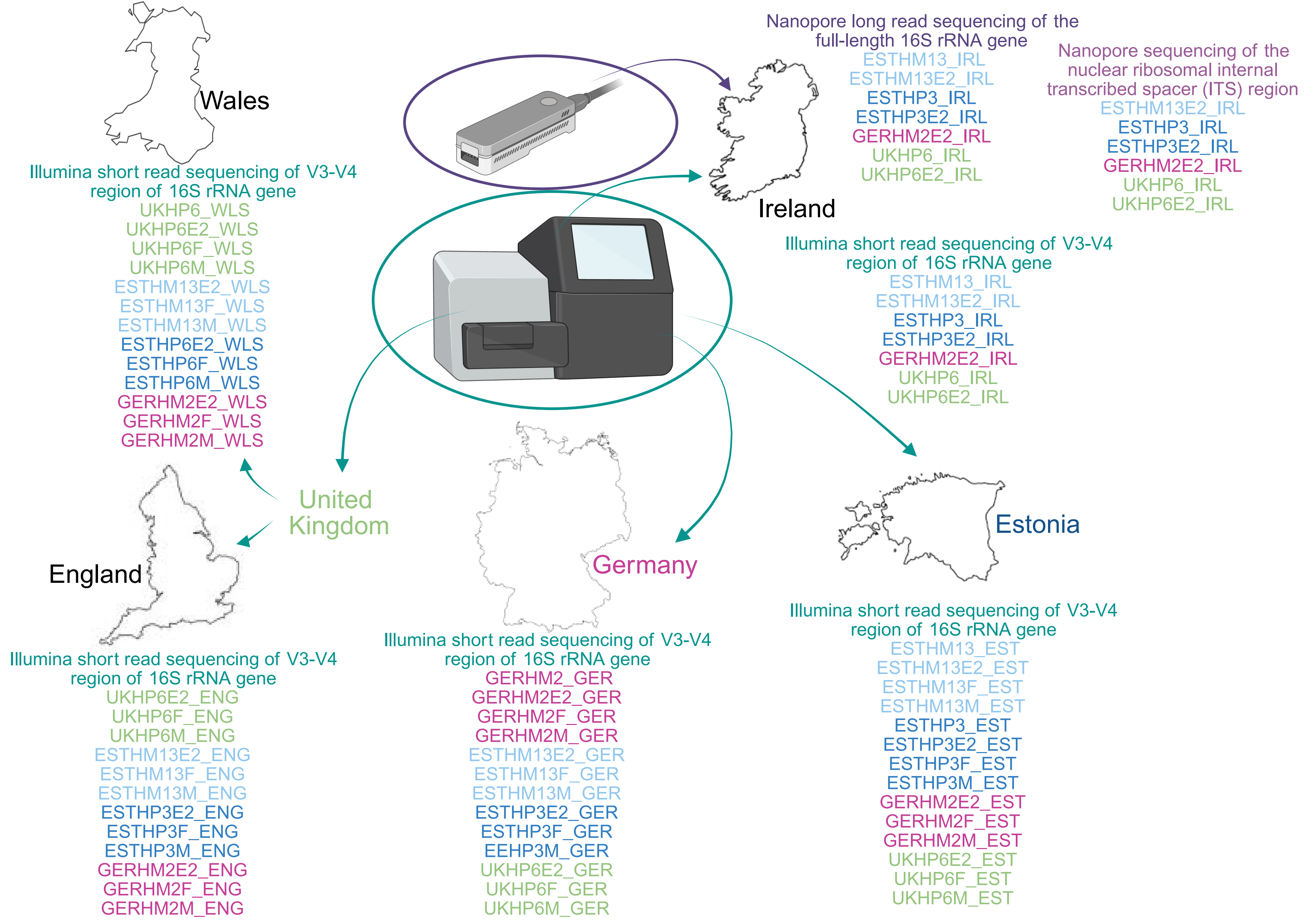
Wales
Illumina short read sequencing of V3-V4 region of 16S rRNA gene
UKHP6_WLS
UKHP6E2_WLS
UKHP6F_WLS
UKHP6M_WLS
ESTHM13E2_WLS
ESTHM13F_WLS
ESTHM13M_WLS
ESTHP6E2_WLS
ESTHP6F_WLS
ESTHP6M_WLS
GERHM2E2_WLS
GERHM2F_WLS
GERHM2M_WLS
Nanopore long read sequencing of the full-length 16S rRNA gene
ESTHM13_IRL
ESTHM13E2_IRL
ESTHP3_IRL
ESTHP3E2_IRL
GERHM2E2_IRL
UKHP6_IRL
UKHP6E2_IRL
Nanopore sequencing of the nuclear ribosomal internal transcribed spacer (ITS) region
ESTHM13E2_IRL
ESTHP3_IRL
ESTHP3E2_IRL
GERHM2E2_IRL
UKHP6_IRL
UKHP6E2_IRL
Ireland
Illumina short read sequencing of V3-V4 region of 16S rRNA gene
ESTHM13_IRL
ESTHM13E2_IRL
ESTHP3_IRL
ESTHP3E2_IRL
GERHM2E2_IRL
UKHP6_IRL
UKHP6E2_IRL
United Kingdom
England
Germany
Estonia
Illumina short read sequencing of V3-V4 region of 16S rRNA gene
UKHP6E2_ENG
UKHP6F_ENG
UKHP6M_ENG
ESTHM13E2_ENG
ESTHM13F_ENG
ESTHM13M_ENG
ESTHP3E2_ENG
ESTHP3F_ENG
ESTHP3M_ENG
GERHM2E2_ENG
GERHM2F_ENG
GERHM2M_ENG
Illumina short read sequencing of V3-V4 region of 16S rRNA gene
GERHM2_GER
GERHM2E2_GER
GERHM2F_GER
GERHM2M_GER
ESTHM13E2_GER
ESTHM13F_GER
ESTHM13M_GER
ESTHP3E2_GER
ESTHP3F_GER
EEHP3M_GER
UKHP6E2_GER
UKHP6F_GER
UKHP6M_GER
Illumina short read sequencing of V3-V4 region of 16S rRNA gene
ESTHM13_EST
ESTHM13E2_EST
ESTHM13F_EST
ESTHM13M_EST
ESTHP3_EST
ESTHP3E2_EST
ESTHP3F_EST
ESTHP3M_EST
GERHM2E2_EST
GERHM2F_EST
GERHM2M_EST
UKHP6E2_EST
UKHP6F_EST
UKHP6M_EST

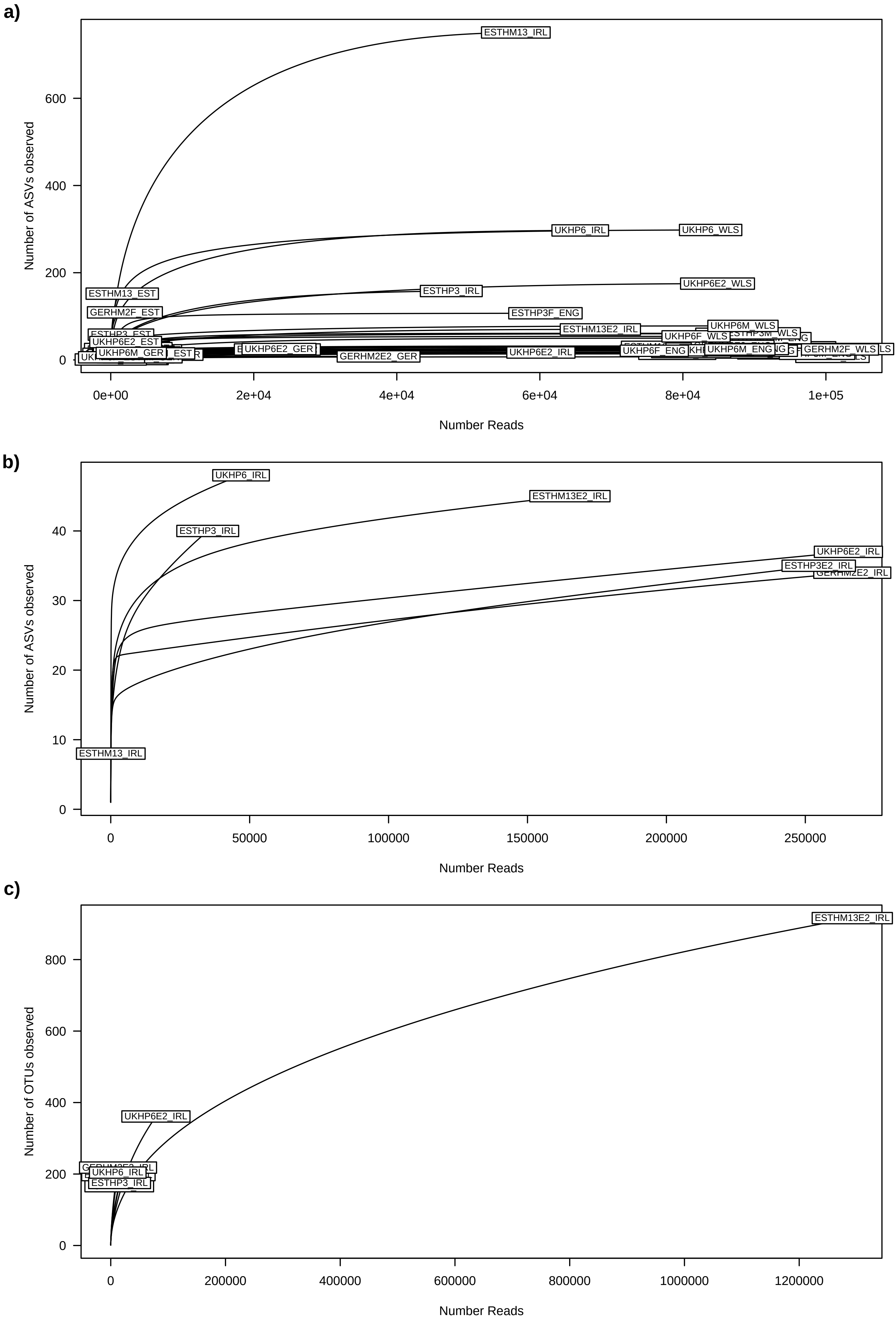

a)
Number of ASVs observed
Number Reads
0
200
400
600
0e+00
2e+04
4e+04
6e+04
8e+04
1e+05
ESTHM13_IRL
UKHP6_IRL
UKHP6_WLS
ESTHM13_EST
ESTHP3_IRL
UKHP6E2_WLS
GERHM2F_EST
ESTHP3F_ENG
ESTHM13E2_IRL
UKHP6M_WLS
ESTHP3_EST
UKHP6F_WLS
UKHP6E2_EST
UKHP6E2_GER
UKHP6M_GER
GERHM2E2_GER
UKHP6E2_IRL
UKHP6F_ENG
UKHP6M_ENG
GERHM2F_WLS
b)
Number of ASVs observed
Number Reads
0
10
20
30
40
0
50000
100000
150000
200000
250000
UKHP6_IRL
ESTHM13E2_IRL
ESTHP3_IRL
UKHP6E2_IRL
ESTHP3E2_IRL
ESTHM13_IRL
c)
Number of OTUs observed
Number Reads
0
200
400
600
800
0
200000
400000
600000
800000
1000000
1200000
ESTHM13E2_IRL
UKHP6E2_IRL
UKHP6_IRL
ESTHP3_IRL

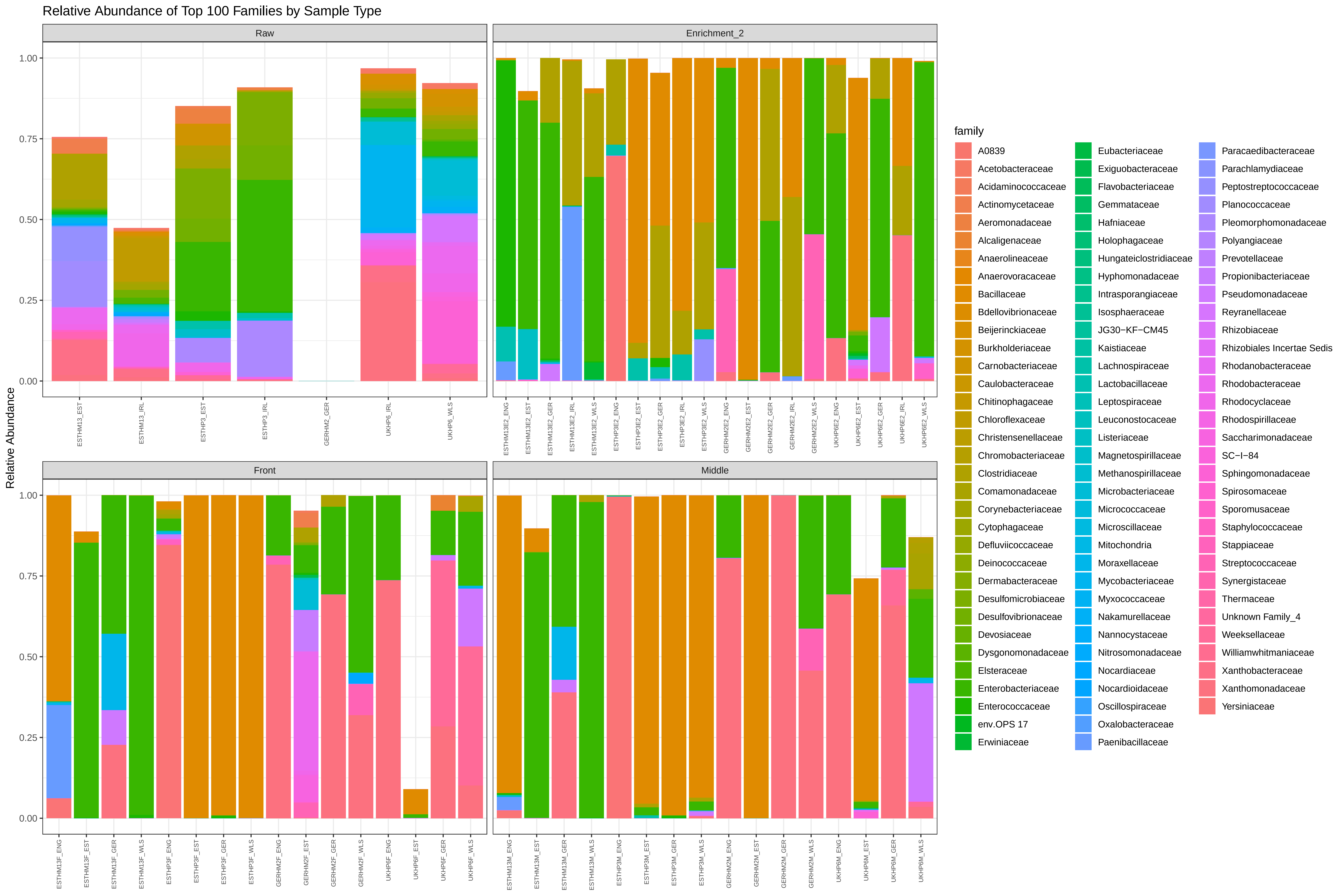

Relative Abundance of Top 100 Families by Sample Type
Raw
Enrichment_2
Front
Middle
Relative Abundance
1.00
0.75
0.50
0.25
0.00
ESTHM13_EST
ESTHM13_IRL
ESTHP3_EST
ESTHP3_IRL
GERHM2_GER
UKHP6_IRL
UKHP6_WLS
ESTHM13E2_ENG
ESTHM13E2_EST
ESTHM13E2_GER
ESTHM13E2_IRL
ESTHM13E2_WLS
ESTHP3E2_ENG
ESTHP3E2_EST
ESTHP3E2_GER
ESTHP3E2_IRL
ESTHP3E2_WLS
GERHM2E2_ENG
GERHM2E2_EST
GERHM2E2_GER
GERHM2E2_IRL
GERHM2E2_WLS
UKHP6E2_ENG
UKHP6E2_EST
UKHP6E2_GER
UKHP6E2_IRL
UKHP6E2_WLS
ESTHM13F_ENG
ESTHM13F_EST
ESTHM13F_GER
ESTHM13F_WLS
ESTHP3F_ENG
ESTHP3F_EST
ESTHP3F_GER
ESTHP3F_WLS
GERHM2F_ENG
GERHM2F_EST
GERHM2F_GER
GERHM2F_WLS
UKHP6F_ENG
UKHP6F_EST
UKHP6F_GER
UKHP6F_WLS
ESTHM13M_ENG
ESTHM13M_EST
ESTHM13M_GER
ESTHM13M_WLS
ESTHP3M_ENG
ESTHP3M_EST
ESTHP3M_GER
ESTHP3M_WLS
GERHM2M_ENG
GERHM2M_EST
GERHM2M_GER
GERHM2M_WLS
UKHP6M_ENG
UKHP6M_EST
UKHP6M_GER
UKHP6M_WLS
family
A0839
Acetobacteraceae
Acidaminococcaceae
Actinomycetaceae
Aeromonadaceae
Alcaligenaceae
Anaerolineaceae
Anaerovoracaceae
Bacillaceae
Bdellovibrionaceae
Beijerinckiaceae
Burkholderiaceae
Carnobacteriaceae
Caulobacteraceae
Chitinophagaceae
Chloroflexaceae
Christensenellaceae
Chromobacteriaceae
Clostridiaceae
Comamonadaceae
Corynebacteriaceae
Cytophagaceae
Defluviicoccaceae
Deinococcaceae
Dermabacteraceae
Desulfomicrobiaceae
Desulfovibrionaceae
Devosiaceae
Dysgonomonadaceae
Elsteraceae
Enterobacteriaceae
Enterococcaceae
env.OPS 17
Erwiniaceae
Eubacteriaceae
Exiguobacteraceae
Flavobacteriaceae
Gemmataceae
Hafniaceae
Holophagaceae
Hungateiclostridiaceae
Hyphomonadaceae
Intrasporangiaceae
Isosphaeraceae
JG30−KF−CM45
Kaistiaceae
Lachnospiraceae
Lactobacillaceae
Leptospiraceae
Leuconostocaceae
Listeriaceae
Magnetospirillaceae
Methanospirillaceae
Microbacteriaceae
Micrococcaceae
Microscillaceae
Mitochondria
Moraxellaceae
Mycobacteriaceae
Myxococcaceae
Nakamurellaceae
Nannocystaceae
Nitrosomonadaceae
Nocardiaceae
Nocardioidaceae
Oscillospiraceae
Oxalobacteraceae
Paenibacillaceae
Paracaedibacteraceae
Parachlamydiaceae
Peptostreptococcaceae
Planococcaceae
Pleomorphomonadaceae
Polyangiaceae
Prevotellaceae
Propionibacteriaceae
Pseudomonadaceae
Reyranellaceae
Rhizobiaceae
Rhizobiales Incertae Sedis
Rhodanobacteraceae
Rhodobacteraceae
Rhodocyclaceae
Rhodospirillaceae
Saccharimonadaceae
SC−I−84
Sphingomonadaceae
Spirosomaceae
Sporomusaceae
Staphylococcaceae
Stappiaceae
Streptococcaceae
Synergistaceae
Thermaceae
Unknown Family_4
Weeksellaceae
Williamwhitmaniaceae
Xanthobacteraceae
Xanthomonadaceae
Yersiniaceae

Supplementary Figure S1. Schematic representation of the sequencing strategies used for each biofilm sample and the location where this sequencing was performed. Coloured font denote the country of origin of the biofilm (see also **Table 1**).

Supplementary Figure S2. Rarefaction curves of the reads obtained from purified gDNA taken from raw, enriched and post-SSCBM biofilms for a) 16S rRNA amplicon-mediated sequencing of the V3-V4 polymorphic region, b) full-length 16S rRNA gene sequencing and c) nuclear ribosomal internal transcribed spacer (ITS) region sequencing.

Supplementary Figure S3 A stacked bar chart plot showing the top 100 abundance families for raw, enriched, front section post-SSCBM and middle section post-SSCBM of the complex biofilms.
